# Supplementary material for: Myogenin promotes myocyte fusion to balance fibre number and size
Source: Nat Commun. 2018 Oct 12;9:4232. doi: 10.1038/s41467-018-06583-6 (PMC6185967; doi:10.1038/s41467-018-06583-6)
Supplement: Supplementary file 2 — Supplementary Information [file 41467_2018_6583_MOESM2_ESM.pdf]

# Supplementary Information PDF

# Myogenin promotes myocyte fusion to balance fibre number and size

**Ganassi et al.**

**Supplementary Table 1** **Ganassi et al.**

## List of used primers

| | Genotyping primers | |
|---|---|---|
| **allele** | **FW 5'-3'** | **REV 5'-3'** |
| *myog* *fh265* | AGAGGCTGCCGAAGGTGGAGAT | TGTTCTCCAAATCTGCACTCACCC |
| *myod* *fh261* | GGACCCCAGGCTTGTTC | GTTGGATCTCGGACTGGA |
| *myog* *kg125* and *myog* *kg128* * | TCAGAAACACCCACAAACGCTCAC | GCAGGCCCAGGGGAGACACT |
| | **qPCR primers** | |
| **gene** | **FW 5'-3'** | **REV 5'-3'** |
| *myog* | TCAGAAACACCCACAAACGCTCAC | GCAGGCCCAGGGGAGACACT |
| *myomaker* | GGACAACTTATTCACAGGGA | TCTGTGATTTTGACAAGCAG |
| *myomixer* | TCTGGTTGTCCGACTCTTCG | GCAGGCCCAGGGGAGACACT |
| *jam3b* | GTTTGAATCAATCGAGCTGT | TATTCTGGGTTTAGTGGTGG |
| *jam2a* | GATATTAATTCACAGCGTCCC | CTTTCTCAGTCTTGAACTCG |
| *actin-b2* | ATCCTTCTTGGGTATGGAAT | GACAATACAGTGTTGGCATA |
| *kirrel3l* | ATGCAAAATGCCATCTACTC | TTTCTCTCTTTCCATCCTCC |
| | **Probe primers** | |
| **gene** | **FW 5'-3'** | **REV 5'-3'** |
| *myomaker* | CAATCGCTCCTCAAAGCTTTCTGAT | GGTAATACGACTCACTATAGGGCTTTTTCCTTCATCTGAG |
| *jam2a* | CCAGCTCTCGTTTTCTCGGAT | GGTAATACGACTCACTATAGGACTCATTGACGGTGTAGGTGG |
| *jam3b* | ATCAGCACCTTGGCAGTCCT | GGTAATACGACTCACTATAGGTTTGTGCCTGAAATCCCCCTC |
| *kirrel3l* | AAGCCATTGTGCCTCGGATT | GGTAATACGACTCACTATAGGGTGGCGTAACGCTGAGAGAA |
| | **ChIP-qPCR primers** | |
| **region** | **FW 5'-3'** | **REV 5'-3'** |
| *mymk* E-box1 | GAGACCGCTGTTTCTAGCCA | GTGGGTTGAGTCTCAGACATTT |
| *mymk* E-box2 | CTGGTCCAGGTCTACGCAAG | AAATGTCTCTCCGTGGCCTG |
| | ***myogenin* :*MyogCDS* -IRES-mGFP6nls cloning primers** | |
| **anneals to** | **FW 5'-3'** | **REV 5'-3'** |
| *pBluescript SK-MG12-ZF-Myogenin* (Weinberg et al., 1996) | GAGCTCGAGCTCATGGAGCTTTTCGAGACCAA | GTCGACGTCGACTTATTTGCTTATGTCCACTG |
| *hsp70-4-myogenin-IRES-mGFP6nls* | CCGGGTACCGGTAGAAAAAATCTAGAGATATCGAGCTCATG | CTGCAGGAATTCTACGAATGTTATTTGTATAGTTCATCCATGC |
| *myogenin:GFP* ( Du et al., 2003) | CATTCGTAGAATTCCTGC | TTTTTTCTACCGGTACCC |

overlaps with *myogenin* :*GFP* Du et al., 2003

* primers were used to amplify DNA fragments of 361 bp, 362 bp and 358 bp from wild-type (wt), *kg128* and *kg125* genomic DNAs (gDNA), respectively. PCR products were then digested with EcoRV (New England Biolabs) and analysed by separation on agarose gel. EcoRV produces 2 fragments (197 bp and 164 bp) in case of wt genotype, 3 fragments in case of heterozygote genotype (for *kg128* , 362, 197 and 165 bp; for *kg125* , 358, 197 and 161 bp) and only 1 undigested fragment in homozygote mutant.

GGTAATACGACTCACTATAGG **T7 sequence for antisense probe synthesis**

**Supplementary Table 2** **Ganassi et al.**

## List of number of fish analysed in each experiment

| Figure | Experiment | Allele | Number of fish analysed<br>Total (mutant) |
|---|---|---|---|
| **1b** | *myogenin* ISH | $myog^{kg125}$ | 27 (5) |
| **1b** | *myogenin* ISH | $myog^{kg128}$ | 53 (14) |
| **1d** | Myogenin IF | $myog^{kg128}$ | 26 (7) |
| **1d** | Myogenin IF | $myog^{kg125}$ | 19 (10) |
| **2b** | sMyHC IF and fibre measurements | $myog^{kg125}$ | 13 (5) |
| **2c** | fMyHC IF | $myog^{kg125}$ | 13 (5) |
| **2d** | F-actin (phallodin) | $myog^{kg125}$ | 26 (5) |
| **2d** | titin IF | $myog^{kg125}$ | 25 (7) |
| **2f-g** | α-actinin IF and sarcomere length measurements | $myog^{kg125}$ | 30 (7) |
| **2h** | AchR α-BTX | $myog^{kg128}$ | 15 (4) |
| **2i** | swim assay | $myog^{kg125}$ | 139 (24) FW / 48 (12) MC |
| **3a-c** | *mylpfa* ISH and somite measurements | $myog^{kg128}$ | indicated in figure |
| **3d-i** | somite measurements on β*actin:EGFP* | $myog^{kg128}$ | indicated in figure |
| **4a-b** | Pax3/7 IF and count | $myog^{kg125}$ | indicated in figure |
| **4c** | fusion assay | $myog^{kg125}$ | 6 (3) |
| **4d-g** | fusion assay | $myog^{kg128}$ | 6 (3) |
| **4i** | rescue assay | $myog^{kg128}$ | Mutants: ctrl 5 - Myog o/e 11 |
| **5a** | *mymk* ISH | $myog^{kg128}$ | 48 (10) |
| **5a** | *jam2a* ISH | $myog^{kg128}$ | 50 (13) |
| **5a** | *jam3b* ISH | $myog^{kg128}$ | 51 (12) |
| **5a** | *kirrel3l* ISH | $myog^{kg128}$ | 20 (7) |
| **5b** | qPCRs | $myog^{kg125}$ | see methods |
| **5c-d** | ChIP-qPCR | wt TL | see methods |
| **6a** | *mymk* ISH | $myog^{kg125}$ | 20 (6) |
| **6b** | *mymk* ISH - CyA | $myog^{kg125}$ | EtOH 4 (4) - CyA 6 (6) |
| **6b** | *mymk* qPCR | $myog^{kg128}$ | see methods |
| **6b** | *mymk* qPCR | $myog^{kg125}$ | see methods |
| **6c** | CyA treatment | $myog^{kg125}$ | EtOH 8 (5) - CyA 10 (4) |
| **7a-b** | Adult fish measurements | $myog^{kg128}$ | indicated in figure |
| **7b** | Adult fish measurements | $myog^{kg125}$ | indicated in figure |
| **7b** | Adult fish measurements | $myog^{fh265}$ | indicated in figure |
| **7c-h** | Adult fish analysis | $myog^{kg128}$ | indicated in figure |
| **7i** | NADH-TR | $myog^{kg128}$ | 6 (3) |
| **8b** | Cell culture Differentiation index | $myog^{kg125}$ | 6 (3) |
| **8c** | Cell culture Fusion index | $myog^{kg125}$ | 6 (3) |
| **8d** | Cell culture nuclei distribution | $myog^{kg125}$ | 6 (3) |
| **S1a** | Myogenin IF quantification | $myog^{kg125}$ | 26 (7) |
| **S1b** | *smyhc1/mylpfa* ISH | $myog^{kg125}$ | 19 (8) |
| **S1b** | *mylpfa* ISH | $myog^{kg125}$ | 13 (5) |
| **S1c** | *smyhc1* ISH | $myog^{kg125}$ | 10 (4) |
| **S1d** | s/fMyHC IF | $myog^{kg125}$ | 21 (9) |
| **S1e** | sMyHC IF | $myog^{kg128}$ | 26 (7) |
| **S1f** | fibre morphology | $myog^{kg125}$ | 23 (7) |
| **S2a** | *smyhc1* ISH | $myog^{kg125}$ | 27 (5) |
| **S2b** | somite measurements | $myog^{kg128}$ | indicated in figure |
| **S2c-e** | somite measurements | $myog^{fh265}$ | indicated in figure |
| **S3a** | fusion assay | $myog^{kg128}$ | 6 (3) |
| **S3b** | fusion analysis | $myog^{kg125}$ | 12 (4) |
| **S3d** | Myog MO | wt TL | 12 controls - 9 MO |
| **S3e** | Myog MO | wt TL | 10 controls - 5 MO |
| **S4b** | rescue assay | $myog^{kg128}$ | Mutants: ctrl 6 - Myog o/e 11 |
| **S4b** | Myog overepression in fast fibres | $myog^{kg128}$ | Sibs: ctrl 5 - Myog o/e 11 |
| **S4e** | Myog overepression in slow fibres | $myog^{kg128}$ | Sibs: ctrl 7 - Myog o/e 9 |
| **S4e** | Myog overepression in slow fibres | $myog^{kg128}$ | Mutants: ctrl 4 - Myog o/e 4 |
| **S5a** | *mymk* ISH | $myog^{fh265}$ | 20 (6) |
| **S5a** | *mymk* ISH | $myod^{fh261}$ | 19 (7) |
| **S5b** | *myog* ISH | $myod^{fh261}$ | 17 (3) |
| **S5c** | *mymk* ISH | $myog^{kg125/?}$ | EtOH 28 (sibs) - CyA 14 (sibs) related to fig 6B |
| **S5d** | *mymk* qPCR | $myog^{kg128}$ | see methods |
| **S5d** | *mymk* qPCR | $myog^{kg125}$ | see methods |
| **S5e** | *mymk* ISH | $myog^{kg128/?}$ | EtOH 28 (6) / CyA 25 (7) |
| **S6a** | Adult fish measurements | $myog^{kg128}$ | indicated in figure |
| **S6b** | entire section measurements | $myog^{kg128}$ | 1 het - 1 mutant |
| **S6c** | Adult fish measurements | $myog^{kg128}$ | indicated in figure |
| **S6d** | Adult fish analysis | $myog^{kg128}$ | indicated in figure |
| **S6e** | Adult fish measurements | $myog^{kg125}$ | indicated in figure |
| **S6f** | Cell culture nuclei distribution | $myog^{kg125}$ | 6 (3) |
| **S6g** | fraction of Desmin+ cells | $myog^{kg125}$ | 6 (3) |

# Supplementary Figure 1

Ganassi et al.

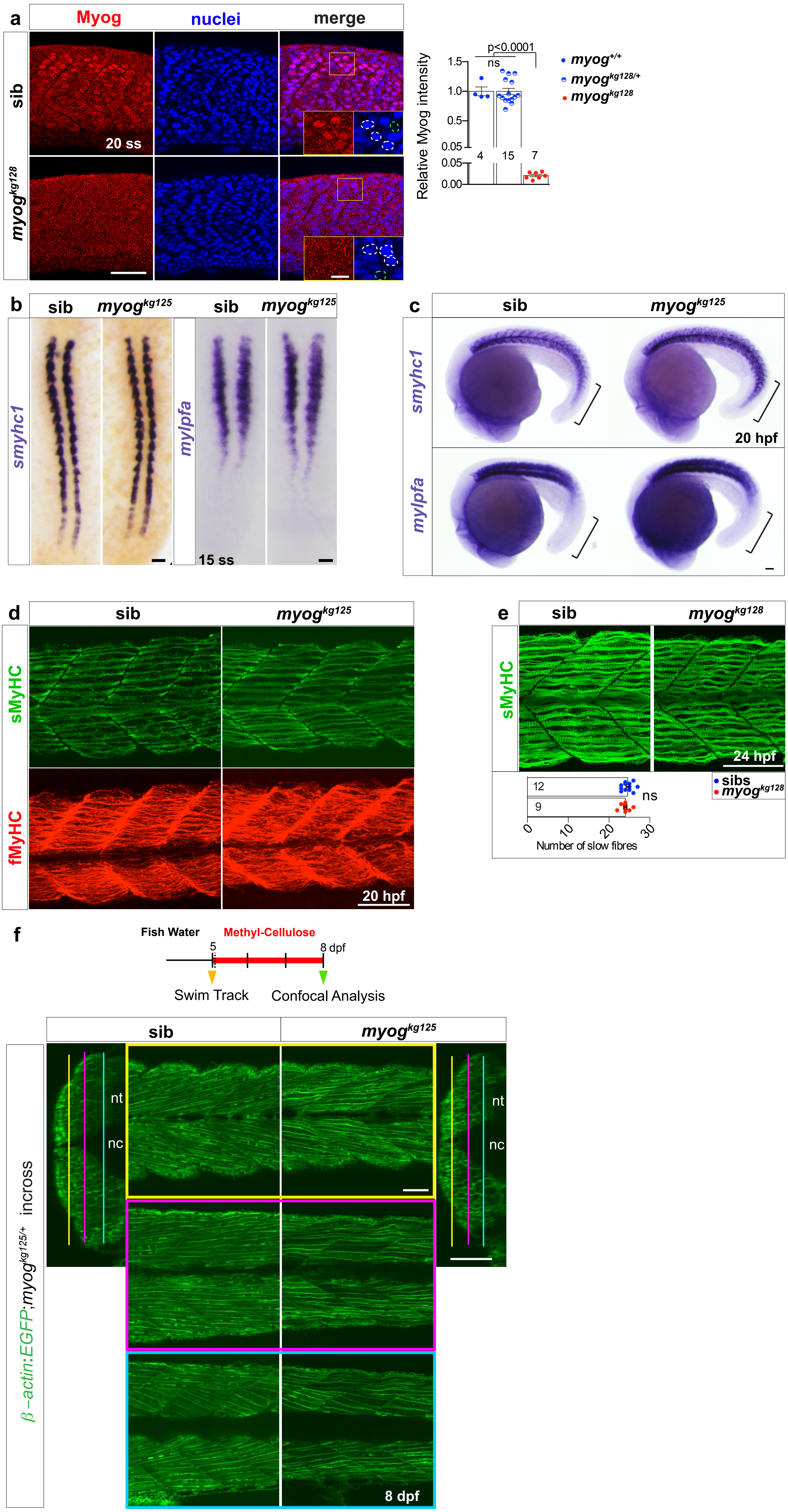

**Supplementary Figure 1 Early myogenesis occurs properly in Myogenin mutant.**

**a**. Immunoreactivity of Myog in nuclei is lost in *myog*$^{kg128}$ mutants at 20 ss. Insets show magnification of boxed areas; dashed lines denote how nuclei (white) or nucleus-free background area (green) was selected for analysis (see Methods). Graph shows quantification of relative myotomal nuclear Myog immunofluorescence; Myog is significantly reduced in mutants but unchanged in heterozygotes compared to wt sibs (summarized data is shown in Fig. 1d). **b.** Dorsal view of ISH for *smyhc1* and *mylpfa* mRNAs at 15 ss embryos from a *myog*$^{kg125/+}$ incross showing that initial differentiation occurs normally despite absence of Myog. **c.** ISH for *smyhc1* and *mylpfa* (brackets highlighting difference in expression) at 20 hpf (22 ss) confirming that *myog*$^{kg125}$ mutant and sib are indistinguishable. **d.** Dual immunodetection using S58 (sMyHC) and EB165 (fMyHC) in 20 hpf embryos from a *myog*$^{kg125/+}$ incross showing that accumulation of slow and fast myosins occurs normally in mutants. **e.** Immunodetection using A4.1025 antibody showing no difference in slow fibre number in mutant compared to sib at 1 dpf (24 hpf). Mean ± SEM, *t*-test. **f.** Fish from *β-actin*:*EGFP*;*myog*$^{kg125/+}$ incross were moved from fish water (FW) to 0.6% methyl-cellulose (MC) solution at 5 dpf and left to grow for 72 hours, then confocally-imaged to assess muscle fibre detachment. Sibling and mutant larvae retain attached muscle throughout the myotome. The locations of three different medio-lateral images per larva within somites 16-18 are shown in transverse sections. Remaining images in a, c-f are lateral views anterior to left, dorsal to top. Replicate numbers are given in Supplementary Table 2. Bars = 50 μm, except for a insets (10 μm).

## Supplementary Figure 2

Ganassi et al.

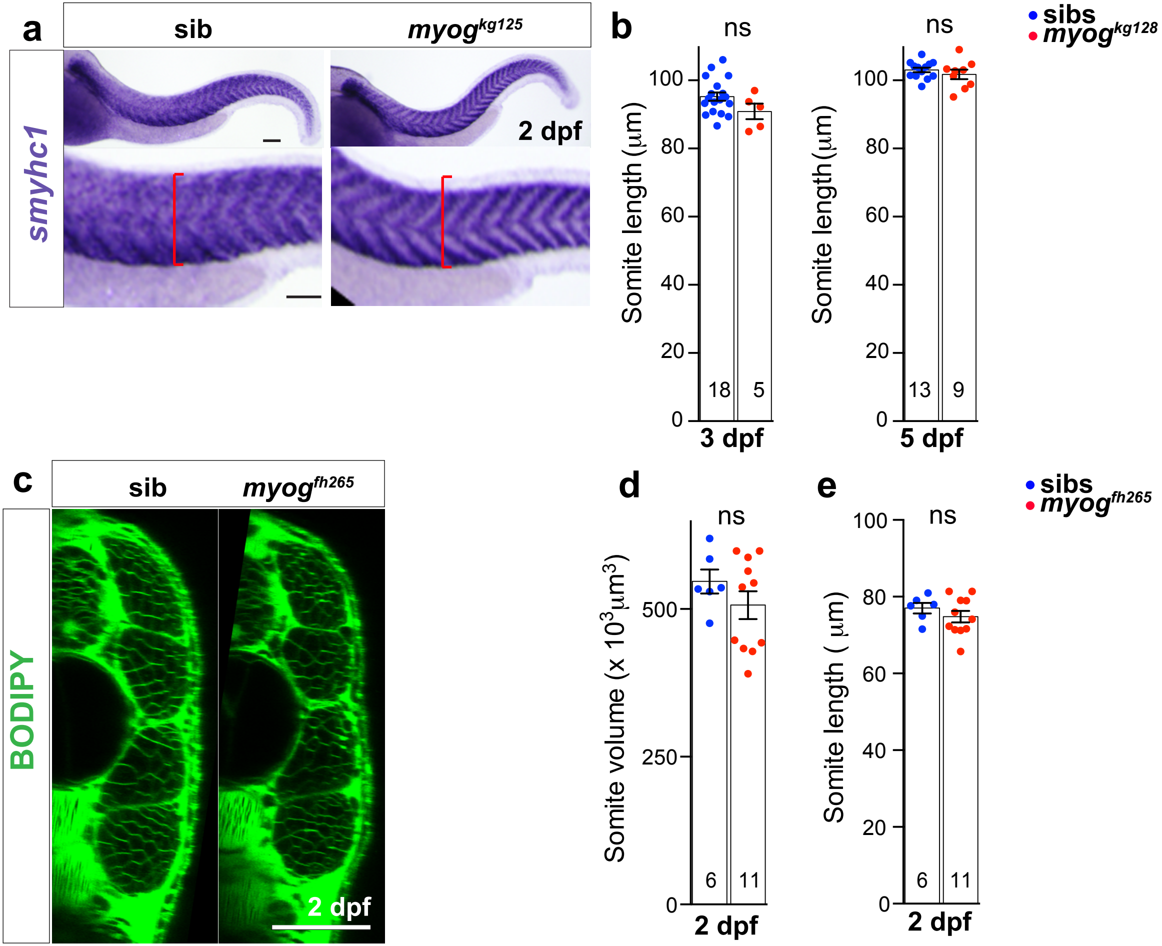

**Supplementary Figure 2** ***Myogenin* mutant has normal *smyhc1* expression and *myog*$^{fh265}$ has no growth defect.**

**a.** ISH shows that at 2 dpf *smyhc1* mRNA level is indistinguishable in *myog*$^{kg125}$ mutant and sibs, but mutant embryos have a reduced extent of somitic muscle (red brackets). **b.** Quantification of somite length at 3 and 5 dpf. **c.** Optical cross sections of *myog*$^{fh265}$ mutants and sibs stained with BODIPY. **d.** Quantitation of somite volume measured in “c”. **e.** Somite length measurement shows that mutants and sibs are comparable. *t*-test, ns= not significant. Replicate numbers are given in Supplementary Table 2. Bars = 50 μm.

**Supplementary Figure 3** Ganassi et al.

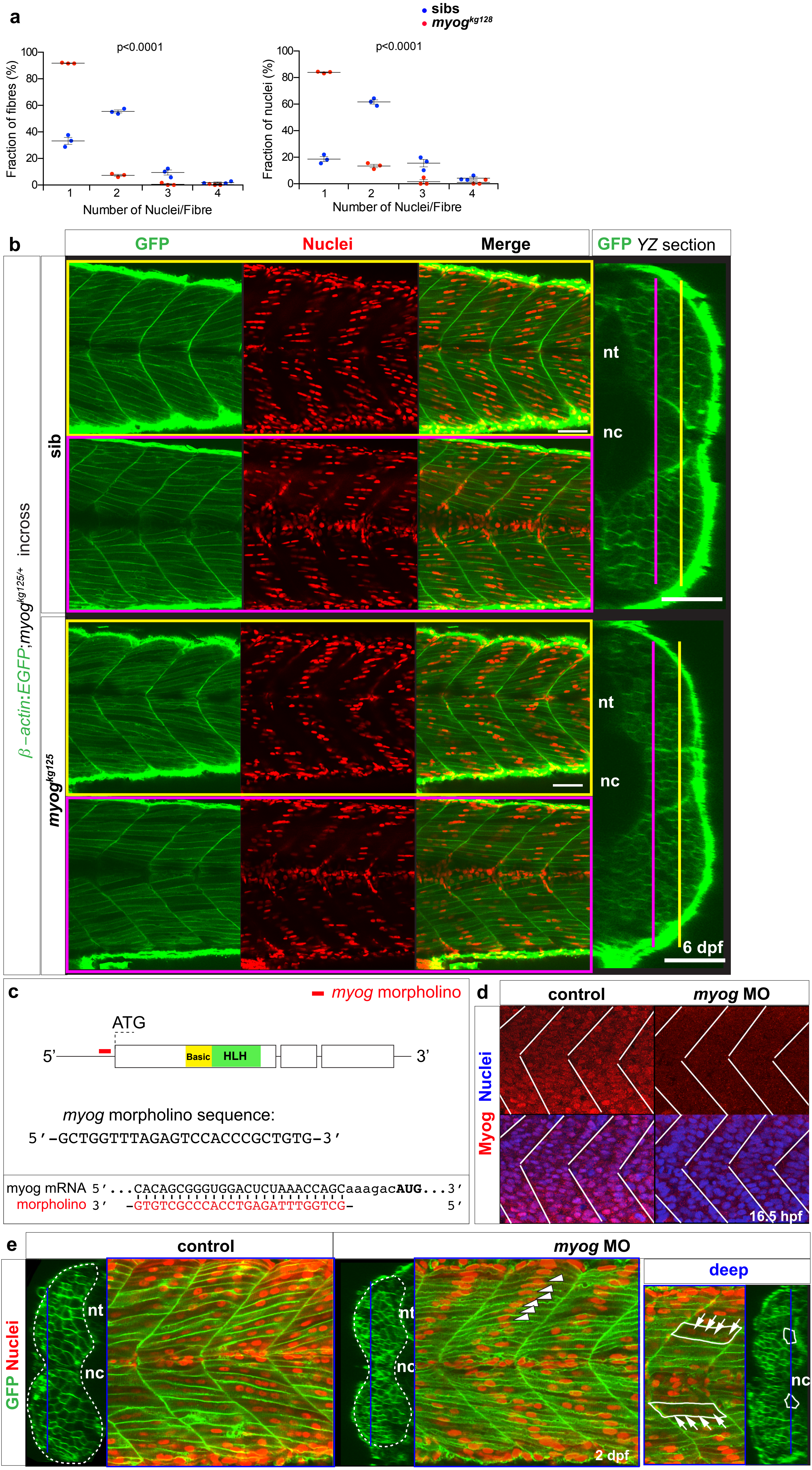

**Supplementary Figure 3 Lack of fusion in *myog*$^{kg125}$ and *myog*$^{kg128}$ persists until 6 dpf and is phenocopied by Myogenin knockdown.**

**a.** Individual quantification of fusion within somite 17 (summarized as pie charts in Fig. 4e-f) showing fraction of fibres with given number of nuclei and fraction of nuclei in fibres with given number of nuclei. Dots represent individual embryos. Mean ± SEM. **b.** Lack of fusion persists in *myog* mutants. GFP staining of *β-actin:EGFP; myog*$^{kg125/+}$ incross at 6 dpf. Nuclei were stained with Hoechst (red). Transverse sections (*YZ*) report position of parasaggital sections (pink and yellow lines). **c.** Schematic of *myog* mRNA showing the position and nucleotide sequence of morpholino (red bold line), which anneals close upstream of ATG start codon. **d.** To knock down Myog, morpholino (MO) was injected at 1-cell stage into *β-actin:EGFP* embryos. Myog immunodetection and Hoechst stained nuclei (blue) at 16.5 hpf showed loss of nuclear Myog immunoreactivity in MO embryos. White lines indicate somite borders. **e.** At 2 dpf, MO and control injected embryos were fixed and incubated with Hoechst to highlight nuclei (red) prior to confocal imaging. As in *myog* mutants, knockdown of Myog led to overabundance of smaller mononucleated muscle fibres and reduced the size of the myotome. Multinucleated fibres remained in deep regions ('deep', right panel) of the myotome. White dashed lines outline somite cross-section, white plain lines outline multinucleated fibres, arrows indicate nuclei within a fibre and blue line indicates the position of the cross sections. nt; neural tube, nc; notochord. Replicate numbers are given in Supplementary Table 2. Bars = 50 μm.

# Supplementary Figure 4

Ganassi et al.

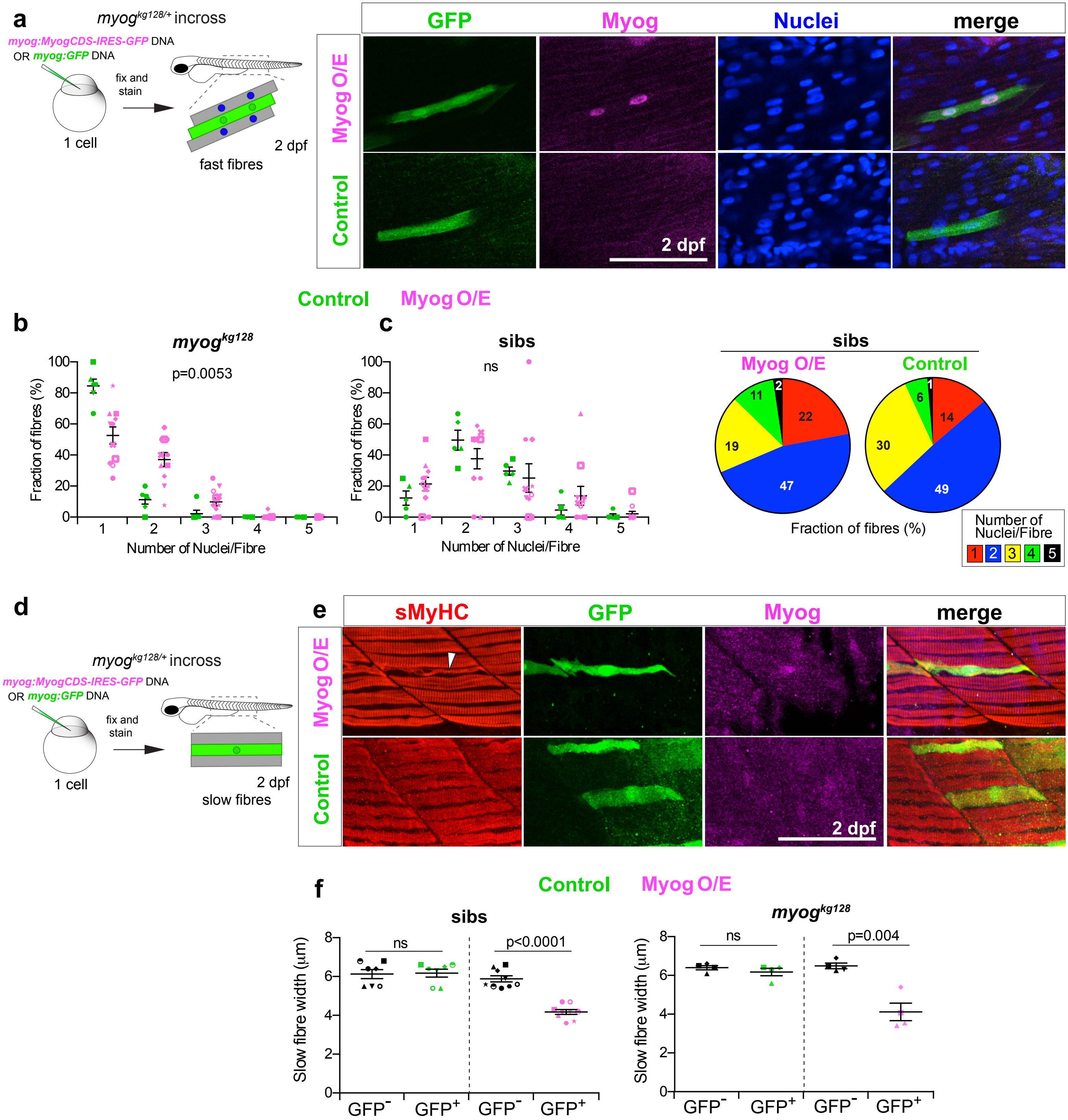

**Supplementary Figure 4 Myog overexpression rescues fast myogenesis in *myog* mutants but does not promote fusion by slow fibres.**

**a.** Quantitative analysis of fusion rescue in a $myog^{kg128/+}$ incross injected at 1-cell stage with DNA encoding *myog:MyogCDS-IRES-GFP* (Myog O/E) or *myog:GFP* (Control) plasmids. Myog and GFP immunodetection and Hoechst stained nuclei (blue) at 2 dpf showed accumulation of Myog in Myog O/E $GFP^{+}$ fibres but not in control $GFP^{+}$ fibres in sibling embryos. **b,c.** Individual quantification of fraction of $GFP^{+}$ fast fibres with the indicated number of nuclei in mutant (b) or sibling (c) embryos from same incross (mutant data also summarized as pie charts in Fig. 4i). Mosaic Myog expression rescues mutant phenotype but does not increase fusion frequency in sibs. Symbol shape represents individual embryos. Mean ± SEM, $\chi^2$ test. **d.** Schematic of analysis (e,f) of *myog:MyogCDS-IRES-GFP* (Myog O/E) or *myog:GFP* (Control) mosaic expression in slow fibres in a $myog^{kg128/+}$ incross. **e.** Slow MyHC (sMyHC, F59), GFP and Myog immunodetection at 2 dpf in a sibling showing accumulation of Myog in a Myog O/E $GFP^{+}$ slow fibre but not in a control $GFP^{+}$ slow fibre. Myog O/E in slow fibres fails to induce fusion but leads to thin fibres with mis-positioned myofibrils (arrowhead). **f.** Quantification of fibre width in mutant ($myog^{kg128}$) or sibling (sibs) embryos mosaically expressing *myog:MyogCDS-IRES-GFP* (Myog O/E, magenta) or *myog:GFP* (Control, green). Symbols represent fibre width of $GFP^{+}$ (magenta or green symbols) or neighbouring $GFP^{-}$ (black symbols) slow fibres within individual embryos, compared by paired *t*-test statistic. Replicate numbers are given in Supplementary Table 2.

Supplementary Figure 5

Ganassi et al.

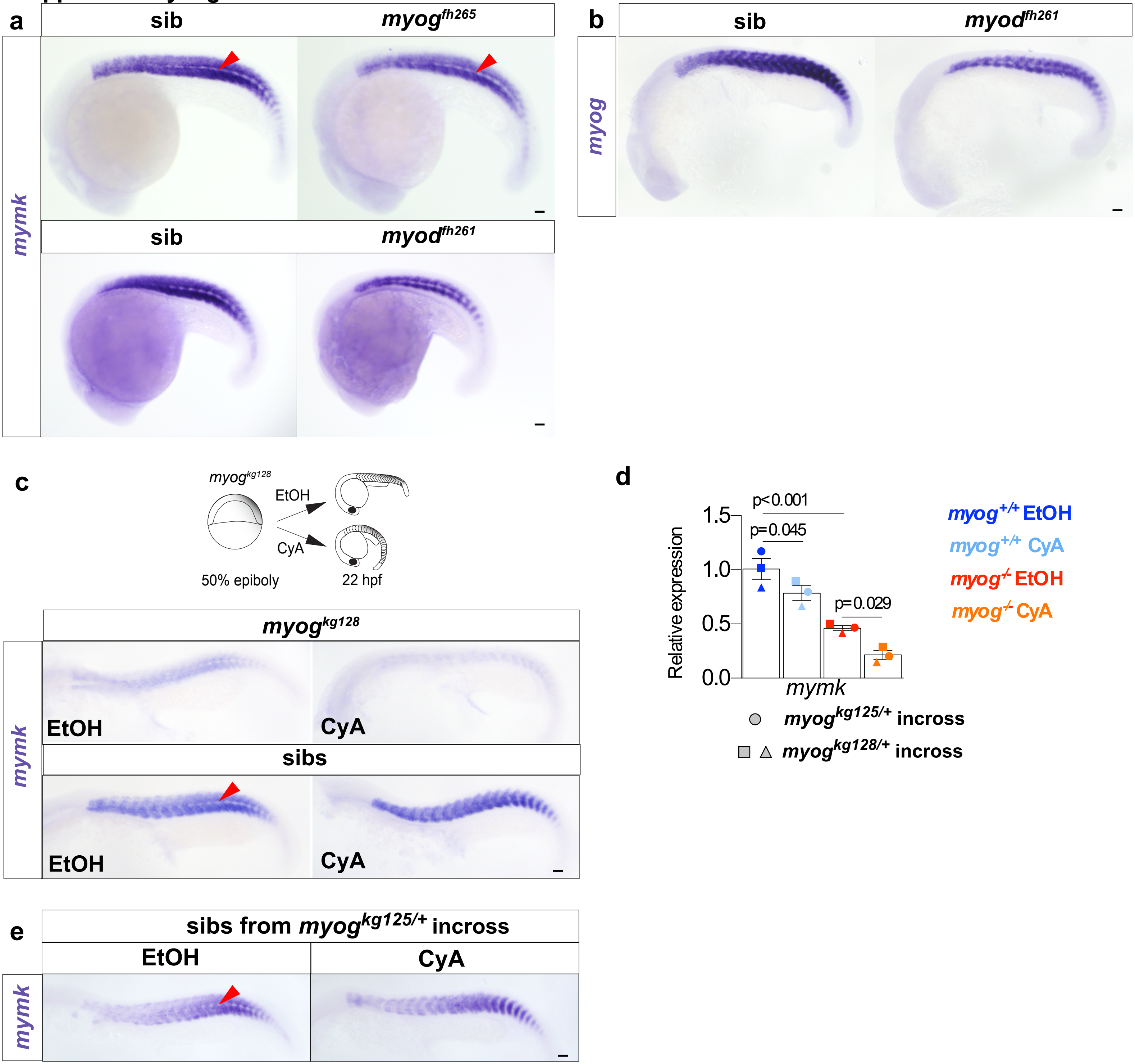

**Supplementary Figure 5 *Myog*$^{fh265}$ and *myod*$^{fh261}$ mutation and cyclopamine treatment have little effect on *myomaker* expression.**

ISH for *mymk* (a,c,e) and *myog* (b) mRNAs. **a.** Expression of *mymk* mRNA is little affected in *myog*$^{fh265}$ and *myod*$^{fh261}$ mutants at 20 hpf. Note lack of expression in mononucleate slow pioneer fibres (arrowheads). **b.** ISH for *myog* showing that mRNA level is reduced in proportion to the loss of fast muscle in *myod*$^{fh261}$ mutant at 20 hpf. **c.** Schematic of CyA (or EtOH vehicle) treatment of embryos from *myog*$^{kg128/+}$ incross. Residual *mymk* mRNA in 22 hpf *myog*$^{kg128}$ mutants is downregulated by CyA-treatment. CyA effectiveness is shown by the loss of unstained slow muscle pioneer cells (arrowheads). **d.** qPCR analysis showing the effect of CyA treatment on both mutant alleles. CyA also significantly reduced *mymk* expression in *myog*$^{+/+}$ embryos. Mean fold change ± SEM from three independent experiments on pooled genotyped embryos from separate lays of *myog*$^{kg125}$ (circles) and *myog*$^{kg128}$ (squares and triangles) analysed on separate days, ANOVA statistic. **e.** ISH for *mymk* on *myog*$^{kg125}$ siblings (related to Fig. 6b). Replicate numbers are given in Supplementary Table 2. Bars = 50 μm.

# Supplementary Figure 6

Ganassi et al.

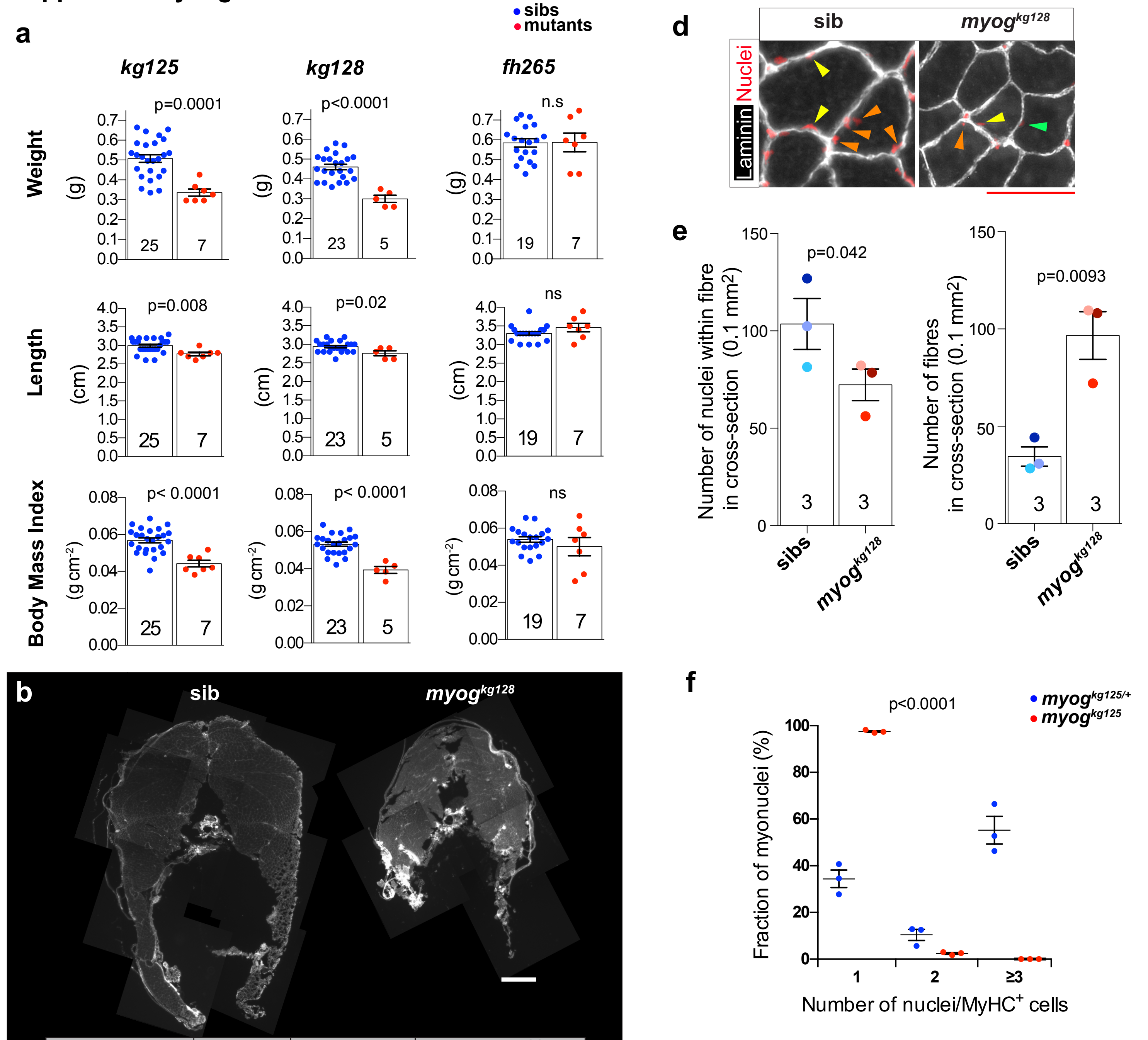


| $myog^{128+/-}$ incross | Area ($mm^2$) | Perimeter (mm) | Total number of fibres in myotome section |
|---|---|---|---|
| sib | 6.1 | 20.7 | 3657 |
| mut | 3.5 | 13.1 | 4097 |
| % Reduction in mut (sib = 100%) | 43 | 36 | |

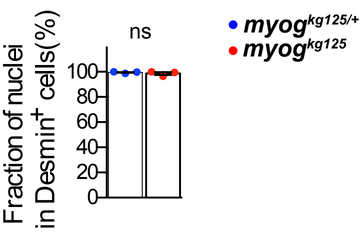


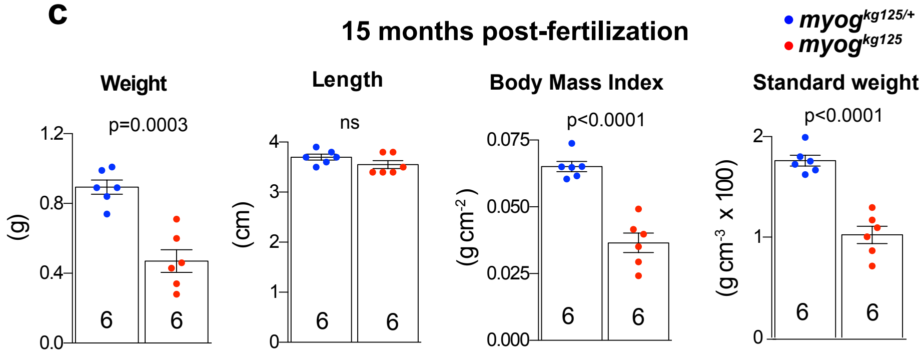

**Supplementary Figure 6 Weight and Body mass index are reduced in *myog* mutants.**

**a.** Weight, length and body mass index of 120 dpf adults derived from *myog*$^{kg128/+}$, *myog*$^{kg125/+}$ and *myog*$^{fh265/+}$ incrosses. Lays were reared as mixed genotypes through nursery at a density of at 50 larvae/3 L tank, decreasing to 17 fish/tank from fingerling stage. Size divergence in sibs did not correlate with genotype (+/+ or +/-), and likely reflects genetic background and feeding rate variation. **b.** Reconstruction of entire adult 120 dpf muscle trunk sections. Table shows total muscle area and perimeter are reduced by 43% and 36% in *myog*$^{kg128}$ mutant adult fish compared to matched sib (sib), whereas fibre number is similar. Bar = 500 µm. **c.** Weight, length, body mass index and standard weight of 15 mpf (months post fertilization) adults derived from *myog*$^{kg125/+}$ incross revealed persistent muscle growth defect in mutants. **d.** Representative images of transverse sections from *myog*$^{kg128}$ mutant and sib. Laminin staining (white) and Hoechst (red) were used to highlight fibres and nuclei. Coloured arrowheads indicate nuclei within single fibres. Bar = 50 µm. **e.** Three fish of each genotype were cryosectioned and nuclei/fibre were scored as nuclear profiles within laminin rings. Total number of nuclei within fibres in each 0.1 mm$^2$ section is mildly reduced, but number of fibres is increased in mutants compared to sibs. Dot colours identify individuals to allow direct comparison between the two analyses. Mean ± SEM, *t*-tests. ns= not significant. **f.** Analysis of adult-derived MPCs from 15 month old *myog*$^{kg125}$ and sibling *myog*$^{kg125/+}$ following 5 days of differentiation. Proportion of nuclei in MyHC$^+$ cells showing fraction of nuclei in cells with given number of nuclei. Same data is shown as pie charts in Fig. 8d. Dots represent data from individual fish. Three fish per genotype (three technical replicate each). Mean ± SEM. **g.** Frequency of Desmin$^+$ cells in mutant is comparable to sib. Three fish per genotype (three technical replicate each). Replicate numbers are given in Supplementary Table 2. Bar = 50 µm (insets = 10 µm).
